# Decreased activity and accelerated apoptosis of neutrophils in the presence of natural polyphenols

**DOI:** 10.2478/v10102-012-0010-9

**Published:** 2012-06

**Authors:** Viera Jančinová, Tomáš Perečko, Juraj Harmatha, Radomír Nosáľ, Katarína Drábiková

**Affiliations:** 1 Institute of Experimental Pharmacology & Toxicology, Slovak Academy of Sciences, SK-84104 Bratislava, Slovakia; 2 Institute of Organic Chemistry and Biochemistry, Academy of Sciences, v.v.i., CZ-16610 Prague, Czech Republic

**Keywords:** neutrophils, oxidative burst, apoptosis, natural polyphenols, resolution of inflammation

## Abstract

Prolonged or excessive formation and liberation of cytotoxic substances from neutrophils intensifies inflammation and the risk of tissue damage. From this perspective, administration of substances which are able to reduce activity of neutrophils and to enhance apoptosis of these cells may improve the therapy of pathological states connected with persistent inflammation. In this short review, neutrophil oxidative burst and apoptosis are presented as potential targets for pharmacological intervention. Effects of natural polyphenols (resveratrol, pterostilbene, pinosylvin, piceatannol, curcumin, N-feruloylserotonin) are summarised, considering the ability of these compounds to affect inflammation and particularly neutrophil activity. The intended neutrophil inhibition is introduced as a part of a new strategy for pharmacological modulation of chronic inflammatory processes, focused on supporting innate anti-inflammatory mechanisms and enhancing resolution of inflammation.

## New strategy for pharmacological modulation of inflammatory processes

Resolution (*i.e.* termination of the defense/beneficial inflammation) has historically been viewed as a passive process, occurring as a result of withdrawal of pro-inflammatory signals. Thus most anti-inflammatory drugs in use suppress mechanisms engaged at the onset and progression of inflammation, but they are not targeted to support natural anti-inflammatory reactions. Only recently has the concept been established that resolution is an active process with a distinct set of chemical mediators (lipoxins, resolvins, protectins), involving decrease in activities of neutrophils and eosinophils, programmed death (apoptosis) of these cells, as well as their clearance from the site of inflammation by macrophages (Kohli & Levy, 2009; Serhan *et al.,* 2007, 2008). An abnormal, ineffective or absent regulation of these processes contributes to cellular dysfunction, tissue damage and to persisting inflammation. Moreover, defective regulation is thought to participate in the pathogenesis of chronic inflammatory diseases, such as asthma, rheumatoid arthritis or chronic obstructive pulmonary disease. Therefore the novel strategy of anti-inflammatory therapy is based upon pharmacological agents capable to enhance the resolution of inflammation (Sawatzky *et al.,* 2006; Serhan *et al.,* 2007; Hallett *et al.,* 2008). Several drugs were found to promote pro-resolution pathways, such as aspirin (stimulates formation of lipoxins), glucocorticoids (activate macrophages and accelerate apoptosis of eosinophils), methotrexate (increases synthesis of endogenous anti-inflammatory mediators), as well as substances modulating neutrophil activity and apoptosis (Yasui & Baba, 2006; Rossi *et al.,* 2007; Filep & El Kebir, 2009).

## Pro-inflammatory activity of neutrophils

Neutrophils (neutrophilic polymorphonuclear leukocytes) represent the body's primary line of defense against invading pathogens. Nevertheless, recently they have been increasingly studied as active participants in the initiation and progression of many pathological states, such as rheumatoid polyarthritis, carcinoma, allergy or ischaemia-reperfusion. All these conditions are generally accompanied by dysregulated, persistent and excessive activation of neutrophils, resulting in damage of adjacent tissues by neutrophil “destructive hardware” – by reactive oxygen species, cytotoxic proteins and proteolytic enzymes (Cascao *et al.*, 2009; Cascao *et al.*, 2010; Fialkow *et al.,* 2007; Wright *et al.,* 2010). In rheumatoid arthritis, neutrophil derived oxidants can induce cartilage degradation, depolymerise hyaluronan and decrease its lubricative properties. Further they can reduce the protective antioxidant and antiproteinase capacity of synovial fluid and thus participate in joint erosion (Cascao *et al.*, 2009; Edwards & Hallett, 1997). Besides, neutrophils are capable to release inflammatory mediators (eicosanoids, chemokines, cytokines), which along with their altered recruitment and delayed apoptosis, have the potential to maintain permanent inflammation (Cascao *et al.*, 2010; Wright *et al.,* 2010). The treatment of diseases associated with chronic inflammation should thus be focused also on neutrophil functions; formation of reactive oxygen intermediates and apoptosis of these cells represent two promising targets for pharmacological intervention.

Box 1 **Factors regulating neutrophil apoptosis**

**Pro-apoptotic factors**

- Bcl-2 (B-cell lymphoma-2) proteins **Bak, Bax, Bid** control integrity of mitochondrial membrane
- Bcl-2 proteins **Bad, Bim** activate Bak/Bax and inhibit antiapoptotic Bcl-2 proteins
- **Cytochrome c**, **APAF1** (apoptotic protease-activating factor-1) activate caspase-9
- **Caspases** (cystein-dependent aspartate-directed proteases); initiate caspases-8 and -9 activate the executioner caspase-3, responsible for cytomorphological alterations
- **TNFα** (tumour necrosis factor-α, high concentrations) activates caspase-8
- **Reactive oxygen intermediates** cause DNA damage, upregulate death receptor clustering and activation of caspase-8
- **Cathepsin D** activates caspase-8 and Bid
- **Calpain 1** regulates Bax and caspase-3 activation
- **Inhibitors of CDK** (cycline-dependent kinase), *e.g.* R-roscovitine, reduce Mcl-1 level

**Anti-apoptotic factors**

- Bcl-2 (B-cell lymphoma-2) proteins **Mcl-1, Bcl-XL, A1** control integrity of mitochondrial membrane
- **XIAP** (X-linked inhibitor of apoptosis protein) inhibits activity of caspases -3 and –9
- **cAMP/protein kinase A** inhibits caspase-8
- **NF-κB** (nuclear factor-κB) increases transcription of survival proteins Mcl-1 and A1
- **p38MAPK** (mitogen-activated protein kinase) inhibits caspases -8 and –3
- **PI3K** (phosphoinositide-3-kinase) inhibits Bad and Bax activation
- **TNFα** (tumour necrosis factor-a, lower concentrations) stimulates expression of A1
- **GM-CSF** (granulocyte macrophage-colony stimulating factor) upregulates antiapoptotic pathways such as PI3K, blocks Bid/Bax redistribution
- **LPS** (lipopolysaccharide) upregulates pro-survival factors Mcl-1 and A1

## Formation of reactive oxygen species

Reactive oxygen species (ROS) are produced in large quantities when neutrophils are stimulated by pro-inflammatory agents or by particles such as bacteria. This process, known as “oxidative burst”, is initiated by the activation of NADPH oxidase (NADPH: nicotine amide adenine dinucleotide phosphate). During this process, the cytosolic *phox* proteins (*phox*: phagocyte oxidase) p47^*phox*^, p67^*phox*^, p40^*phox*^ and Rac2 translocate to the plasma membrane or to membranes of specific granules, where they associate with the membrane-bound components (p22^*phox*^, gp91^*phox*^) to assemble the catalytically active oxidase (El-Benna *et al.*, 2010; Ambruso *et al.,* 2004). A partial association of NADPH oxidase components was observed in neutrophils primed with TNFα, GM-CSF or LPS. This configuration, not sufficient for ROS generation, amplifies the oxidative burst initiated by subsequent stimulation of neutrophils (Sheppard *et al.,* 2005).

Activated NADPH oxidase transfers an electron from NADPH to molecular oxygen, generating superoxide anion. This precursor of other ROS is immediately transformed into hydrogen peroxide (H_2_O_2_), spontaneously or through enzymatic dismutation by superoxide dismutase. Interaction between H_2_O_2_ and superoxide anion can give rise to the hydroxyl radical, one of the most powerful oxidants. Moreover, hydrogen peroxide is a substrate of myeloperoxidase, which catalyses its transformation into highly toxic molecules such as hypochlorous acid, chloramines and tyrosyl radicals (El-Benna *et al.*, 2010; Robinson, 2009). The percentage of particular oxygen metabolites was found to be dependent on the mechanism of neutrophil activation (Takahashi *et al.,* 1991).

Activated neutrophils form and liberate reactive oxygen species both extra- and intracellularly (El-Benna *et al.*, 2010; Karlsson & Dahlgren, 2002). Oxidative burst arising inside neutrophils is much less pronounced and reaches maximum values later than the external ROS formation. The intracellular oxidants fulfill a regulatory role and participate in the initiation of neutrophil apoptosis (Luo & Loison, 2008; Witko-Sarsat *et al.,* 2011). The substantial part of reactive oxygen species is formed at neutrophil plasma membranes and is liberated extracellularly or into phagosomes. These radicals are involved in the elimination of pathogens, however their overproduction may result in damage of surrounding tissues. Confirmation of the destructive role of radicals in pathological states associated with persistent inflammation (Halliwell & Whiteman, 2004; Lonkar & Dedon, 2011) initiated an intensive search for substances with antioxidant activity. Since the optimum therapy is expected to minimise tissue damage without reduction of the physiological function of neutrophils, separate analysis of extra- and intracellular effects of antioxidants is of particular importance. The chemiluminescence method, based on different abilities of luminol and isoluminol to cross biological membranes and to interact with radicals inside cells, represents an effective tool for such differentiation (Drábiková *et al.,* 2006, 2009).

## Neutrophil apoptosis

Apoptosis is a complex physiological mechanism in which a cell undergoes programmed death as a result of withdrawal of survival factors and/or of exposure to pro-apoptotic signals. It represents a sensitive and highly regulated process (Box 1), which includes mitochondrial membrane permeabilisation, followed by the release of cytochrome c and other pro-apoptotic proteins into the cytosol, gradual activation of caspases, DNA fragmentation, chromatin condensation, loss of membrane asymmetry and formation of apoptotic bodies. In contrast to necrosis, apoptosis saves the integrity of neutrophil membranes and prevents the discharge of cytotoxic and proteolytic contents into the surrounding tissues (Luo & Loison, 2008; Witko-Sarsat *et al.,* 2011; Hallett *et al.,* 2008). Alterations in plasma membrane (*e.g.* externalisation of phosphatidylserine) facilitate the recognition and clearance of apoptotic neutrophils by macrophages, resulting in safe removal of these cells from the site of inflammation. Moreover, on recognising apoptotic neutrophils, macrophages are programmed to upregulate the production of anti-inflammatory mediators such as transforming growth factor (TGF)-β and interleukin (IL)-10, resulting in reduction of the inflammatory process (Duffin *et al.,* 2010; Nussbaum & Shapira, 2011). Due to these facts, better comprehension of neutrophil apoptosis might lead to novel therapeutic strategies designed to enhance resolution of inflammation without tissue damage (Witko-Sarsat *et al.,* 2011). At present, a great deal of substances directing neutrophils to apoptosis are tested *in vitro* as well as under conditions of experimental inflammation (Hallett *et al.*, 2008; Hu *et al.,* 2005; Sawatzky *et al.,* 2006; Derouet *et al.,* 2006; Serhan *et al.,* 2007; Jagetia & Aggarwal, 2007). The most intensively examined substances have been inhibitors of nuclear factor-κB, compounds modifying the expression of pro- and anti-apoptotic Bcl-2 (B-cell lymphoma-2) proteins or PI3K (phosphoinositide-3-kinase) and CDK (cycline-dependent kinase) inhibitors.

## Neutrophils as potential targets for pharmacological intervention

**Figure 1:**
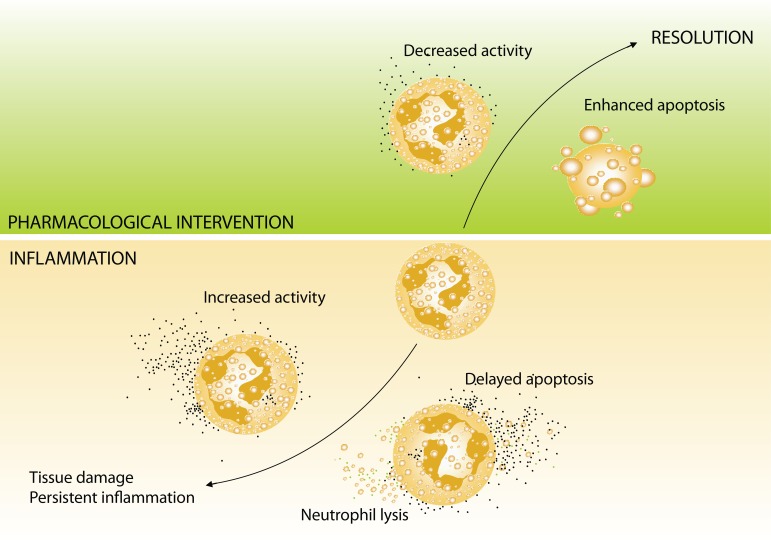
Scheme illustrating the impact of neutrophils on the fate of inflammatory reaction. Prolonged or excessive formation and liberation of cytotoxic substances from neutrophils, along with delayed apoptosis of these cells, intensify inflammation and the risk of tissue damage. Pharmacological intervention, accelerating apoptosis and reducing activities of neutrophils, leads to resolution and contributes to termination of inflammatory reaction.

Prolonged or excessive formation and liberation of cytotoxic substances from neutrophils, accompanied by delayed apoptosis of these cells, intensify inflammation and the risk of tissue damage (Figure 1). From this perspective, the pharmacological intervention capable to enhance the resolution of inflammation through modulation of its important inputs – activity and apoptosis of neutrophils – represents a prospective alternative. To date, the intended neutrophil inhibition has not been involved in the therapy of pathological states connected with persistent inflammation. Since this therapy is long-lasting and often accompanied with many adverse reactions, co-application of substances which are able to reduce the activity of neutrophils and/or to enhance apoptosis of these cells may lead to its increased effectiveness and reduced toxicity. In this regard, the group of natural polyphenols could provide appropriate candidates for such a combined therapy.

## Natural polyphenols and their effects on neutrophils

Phenolic compounds of plant origin integrate a large group of plant secondary metabolites with remarkably rich structural variations. They are generally characterised as aromatic compounds, possessing one or more hydroxyls attached directly to the aromatic (phenolic) moiety of the molecule. The most common types of plant phenols involve phenolic acids, coumarins, stilbenes, flavonoids, lignans, condensed tannins and lignins (Harmatha *et al.,* 2011). In this review, effects of two derivatives of ferulic acid (curcumin, N-feruloylserotonin) and four stilbenes (resveratrol, pterostilbene, pinosylvin, piceatannol) are summarised, considering the ability of these compounds to affect inflammation and particularly neutrophil activity.

Besides their broad structural variability, natural polyphenols are characterised by a great variety of biological effects. Beneficial anti-inflammatory activities, most completely examined for resveratrol and curcumin (Table 1), have been attributed to the capacity of plant phenols to prevent activation of nuclear factor-kappa B and the subsequent overexpression of pro-inflammatory mediators – cytokines, adhesion molecules, cyclooxygenase-2, 5-lipoxygenase, myeloperoxidase or inducible nitric oxide synthase. Minor attention has been concentrated on the effect of polyphenols on the activity of neutrophils (Table 2). The existing results suggest that plant polyphenols can control neutrophil activity by multiple mechanisms and therefore may be more efficient than synthetic inhibitors directed against one particular enzyme or receptor (Burgos *et al.,* 2009).

**Table 1:** Mechanisms of the anti-inflammatory activity of natural polyphenols.

Compound		References
Resveratrol	↓inflammatory biomarkers (e.g. TNFα, 5-lipoxygenase, COX-1, COX-2, TXB_2_, iNOS, CRP), expression of angiogenic and metastatic gene products (e.g. MMPs, VEGF, cathepsin D, and ICAM-1), ↓activity protein kinases (*e.g.* src, PI3K, JNK, and AKT), ↓expression of antiapoptotic gene products (e.g. Bcl-2, Bcl-X_L_, XIAP and survivin), ↓adenosine nucleotide secretion from activated platelets, ↓IL-8, GM-CSF, activation of NF-κB, ↑antioxidant enzymes (e.g. catalase, superoxide dismutase and hemoxygenase-1), antioxidant activity	Alarcón de la Lastra & Villegas,^2005^; Haricumar & Aggarwal, 2008; Anonym, 2010
Pterostilbene	↓activation of NF-κB, ↓COX-1, ↓COX-2, ↓iNOS ↓production of pro-inflammatory mediators (PGE_2_, TNFα), antioxidant activity	Perečko *et al.,* 2010; Cichocki *et al.,* 2008; Remsberg *et al.,* 2008; Paul *et al.,* 2009; Anonym, 2010;
Pinosylvin	antioxidant activity, ↓activation of NF-κB, ↓production of pro-inflammatory mediators, ↓COX-2 and iNOS expression	2006; Lee *et al.,* 2006; Park *et al.,* 2005; Park *et al.,* 2011
Piceatannol	↓Syk, ↓COX-2, ↓iNOS, antioxidant activity, ↓MPO, ↓PGE_2_, ↓pro-inflammatory cytokines	2006; Son *et al.,* 2010; Kim *et al.,* 2008
Curcumin	↓activation of NF-κB, ↓overexpression of inflammatory cytokines and adhesion molecules, ↓activity of COX-2, iNOS and LOX, antioxidant activity	Jurenka, 2009; Srivastava *et al.,* 2011; Aggarwal & Sung, 2009
N-feruloyl serotonin	antioxidant activity, ↓LDL oxidation, ↓activation of caspase-3, ↓ROS-dependent adhesion, ↓migration of monocytes to endothelial cells, ↓activation of NF-κB	Piga 2009; Piga *et al.,* 2010

Abbreviations used: **AKT**: protein kinase B; **Bcl-2**: B-cell lymphoma 2; **Bcl-X**_**L**_: B-cell lymphoma-extra large; **COX**: cyclooxygenase; **CRP**: C-reactive protein; **GM-CSF**: granulocyte-macrophage colony-stimulating factor; **ICAM**: intracellular adhesion molecule; **IL-8:** interleukin 8; **iNOS**: inducible nitric oxide synthase; **JNK:** c-Jun N-terminal kinase; **LDL**: low density lipoprotein; **LOX**: lipoxygenase; **MAC-1**: macrophage-1 antigen; **MAPK:** mitogen-activated protein kinase; **MMP**: matrix metalloproteinase; **MPO**: myeloperoxidase; **NF-κB**: nuclear factor kappa B; **NO**: nitric oxide; **PGE**_**2**_: prostaglandin E_2_; **PI3K**: phosphoinositide-3 kinase; **ROS**: reactive oxygen species; **Src**: non-receptor tyrosine kinases; **Syk**: spleen tyrosine kinase; **TNFα**: tumour necrosis factor alpha; **TXB**_**2**_: thromboxane B_2_; **VCAM-1**: vascular cell adhesion protein; **VEGF**: vascular endothelial growth factor; **XIAP**: X-linked inhibitor of apoptosis protein

**Table 2:** Effects of natural polyphenols on neutrophils.

Compound		References
Resveratrol	↓superoxide anion, ↓hypochlorous acid, ↓chemotaxis ↓5-LOX, ↓myeloperoxidase, ↓ROS formation ↓expression ICAM-1, VCAM-1, MAC-1, β2-integrin ↓activity protein kinases (MAPK, JNK) ↓elastase, ↓β-glucuronidase, ↓chemotaxis, ↓NO production	Perečko *et al.,* 2008; Alarcón de la Lastra & Villegas, 2005; Cavallaro *et al.,* 2003; Adams *et al.,* 2005; Kohnen *et al.,* 2007
Pterostilbene	↓ROS formation	Perečko *et al.,* 2008; Perečko *et al.,* 2010
Pinosylvin	↓5-LOX, ↓ROS formation	Perečko *et al.,* 2008; Adams *et al.,* 2005
Piceatannol	↓Syk, ↓phagocytosis and adhesion, ↑apoptosis, ↓TNFα, ↓PGE_2_,↓IL-8, ↓ROS production, ↓p40phox phosphorylation	Ennaciri & Girard, 2009; Richard *et al.,* 2005; Jančinová *et al.,* 2011a
Curcumin	↓aggregation, ↓ROS production, ↑apoptosis, ↓chemotaxis, ↓protein kinase C activation, ↓5-LOX	Srivastava *et al.,* 2011; Jančinová *et al.,* 2009; Jančinová *et al.,* 2011; Prasad *et al.,* 2004
N-feruloyl serotonin	↓ROS production, ↓protein kinase C activation	Nosáľ *et al.,* 2011; Nosáľ *et al.,* 2010

Abbreviations used: *see Table 1*.
